# TFE3 and TFEB-rearranged renal cell carcinomas: an immunohistochemical panel to differentiate from common renal cell neoplasms

**DOI:** 10.1007/s00428-022-03380-x

**Published:** 2022-08-18

**Authors:** Anna Caliò, Stefano Marletta, Matteo Brunelli, Serena Pedron, Sofia Canete Portillo, Diego Segala, Elena Bariani, Stefano Gobbo, George Netto, Guido Martignoni

**Affiliations:** 1 Department of Diagnostic and Public Health, Section of Pathology, University of Verona, Verona, Italy; 2 Department of Pathology, University of Alabama at Birmingham, Birmingham, AL USA; 3 Department of Molecular and Translational Medicine, Section of Pathology, University-Spedali Civili of Brescia, Brescia, Italy; 4 Department of Translational Medicine, University of Ferrara, Ferrara, Italy; 5 Department of Pathology, Pederzoli Hospital, Peschiera del Garda, Italy

**Keywords:** Translocation renal cell carcinoma, Cathepsin K, Immunohistochemistry, TFE3, TFEB, Renal cell carcinoma

## Abstract

TFE3/TFEB-rearranged renal cell carcinomas are characterized by translocations involving *TFE3* and *TFEB* genes. Despite the initial description of typical morphology, their histological spectrum is wide, mimicking common subtypes of renal cell tumors. Thus, the diagnosis is challenging requiring the demonstration of the gene rearrangement, usually by FISH. However, this technique is limited in most laboratories and immunohistochemical TFE3/TFEB analysis is inconsistent. We sought to identify a useful immunohistochemical panel using the most common available markers to recognize those tumors. We performed an immunohistochemical panel comparing 27 TFE3-rearranged and 10 TFEB-rearranged renal cell carcinomas to the most common renal cell tumors (150 clear cell, 100 papillary, 50 chromophobe renal cell carcinomas, 18 clear cell papillary renal cell tumors, and 50 oncocytomas). When dealing with neoplasms characterized by cells with clear cytoplasm, CA9 is a helpful marker to exclude clear cell renal cell carcinoma. GATA3, AMACR, and CK7 are useful to rule out clear cell papillary renal cell tumor. CK7 is negative in TFE3/TFEB-rearranged renal cell carcinoma and positive in papillary renal cell carcinoma, being therefore useful in this setting. Parvalbumin and CK7/S100A1 respectively are of paramount importance when TFE3/TFEB-rearranged renal cell carcinoma resembles oncocytoma and chromophobe renal cell carcinoma. Moreover, in TFEB-rearranged renal cell carcinoma, cathepsin K and melanogenesis markers are constantly positive, whereas TFE3-rearranged renal cell carcinoma stains for cathepsin K in roughly half of the cases, HMB45 in 8% and Melan-A in 22%. In conclusion, since TFE3/TFEB-rearranged renal cell carcinoma may mimic several histotypes, an immunohistochemical panel to differentiate them from common renal cell tumors should include cathepsin K, CA9, CK7, and parvalbumin.

**Supplementary Information:**

The online version contains supplementary material available at 10.1007/s00428-022-03380-x.

## Introduction

Molecular classification of renal cell carcinoma has been evolving in the last decades, with emerging of new entities and new genetic characteristics. The forthcoming Word Health Organization (WHO) includes TFE3-rearranged renal cell carcinoma and TFEB-rearranged renal cell carcinoma as separate entities differentiating from the previous one in which the term “MiT family translocation renal cell carcinoma” encompassed both tumor types [1].

Both tumors are frequently discovered during childhood [2]; however, they can affect older patients as well and are both characterized by a translocation involving one of the MiT subfamily transcription factor genes. Despite these common aspects, the two entities present several differences. TFE3-rearranged renal cell carcinoma, as the name indicates, harbors *TFE3* gene translocation which fuses with one of several other genes, such as *ASPL* (*ASPSCR1*), *PRCC*, *SFPQ*, *CLTC*, *PARP14*, *RBM10*, *NONO*, and *MED15* [3–11]. There is a slight female predominance and half of those tumors presented at an advanced stage [10, 12] and behaved aggressively [13]. Histologically, the most typical cases show a mixed papillary and nested pattern with a mixture of cells with clear and granular/oncocytic cytoplasm. Psammoma bodies are often present within the tumor and might be a useful morphologic clue [14]. In TFEB-rearranged renal cell carcinoma *TFEB* gene, located on chromosome 6, most commonly translocated to chromosome 11 where fused with the Alpha (*MALAT1*) gene [15] and for this reason was previously designated as t(6;11) renal cell carcinoma. However, as TFE3-rearranged renal cell carcinoma, other gene fusion partners have been recently detected (*COL21A1*, *CADM2*, *KHDRBS2*, *ACTB*, *EWSR1*, *CTLC*, and *NEAT1*) [16–20], as well as tumors harboring *TFEB* gene amplification despite translocation [21–28]. There is no distinct gender predominance and most of them have an indolent clinical course [13]. Morphologically, the tumors are usually made up of a biphasic proliferation of large epithelioid clear and eosinophilic cells merged with aggregates of smaller cells gathered around spheres of basement membrane-derived material [29, 30].

Although these characteristic patterns can be striking in the classic cases, much more often, those tumors present heterogeneous architectural and cytological features, resembling the common subtypes of renal cell carcinoma. TFE3-rearranged renal cell carcinoma may show a solid, trabecular, or microcystic pattern, mimicking clear cell renal cell carcinoma, papillary renal cell carcinoma, and clear cell papillary renal cell tumor [2, 31–33]. On the other hand, in TFEB-rearranged renal cell carcinoma, a wide range of histological features has been reported, including extensive hyalinization, papillary architecture, clear cell morphology, or eosinophilic appearance mimicking papillary renal cell carcinoma, clear cell renal cell carcinoma, oncocytoma, chromophobe renal cell carcinoma, and epithelioid angiomyolipoma/pure epithelioid PEComa [29, 34, 35].

Due to the wide spectrum of morphology observed, the diagnosis is not straightforward on hematoxylin and eosin slides, and the identification of *TFE3* or *TFEB* gene translocation is required to reach the proper diagnosis. Although it could be argued to use TFE3 and TFEB immunostaining to demonstrate the translocation, the results are inconsistent due to the not infrequent false-positive and false-negative results [36]. For this reason, fluorescent in situ hybridization (FISH) analysis is currently considered the gold standard [36–39]. Recently, the expression of TRIM63 by RNA in situ hybridization (RNA-ISH) assay has been proposed as another diagnostic marker for TFE3 and TFEB-rearranged renal cell carcinoma [40] even if an external validation has not been performed so far. Nevertheless, either FISH or RNA-ISH techniques are limited in most laboratories. Conversely, immunohistochemistry is more commonly available, and pathologists are more familiar to use this tool for diagnostic purposes, especially immunohistochemical markers performed in their practice. However, the range of positive expression of an immunohistochemical marker can be broad and several cutoffs have been reported in the literature and used in clinical practice. For these reasons, in this study, we have performed a detailed immunohistochemical analysis of TFE3 and TFEB-rearranged renal cell carcinoma compared with the most common histotypes of renal cell neoplasms evaluating its usefulness, with different cutoffs, in the differential diagnosis and looking for an immunohistochemical panel using most available markers to recognize those tumors.

## Materials and methods

### Patients and samples

**Fig. 1:**
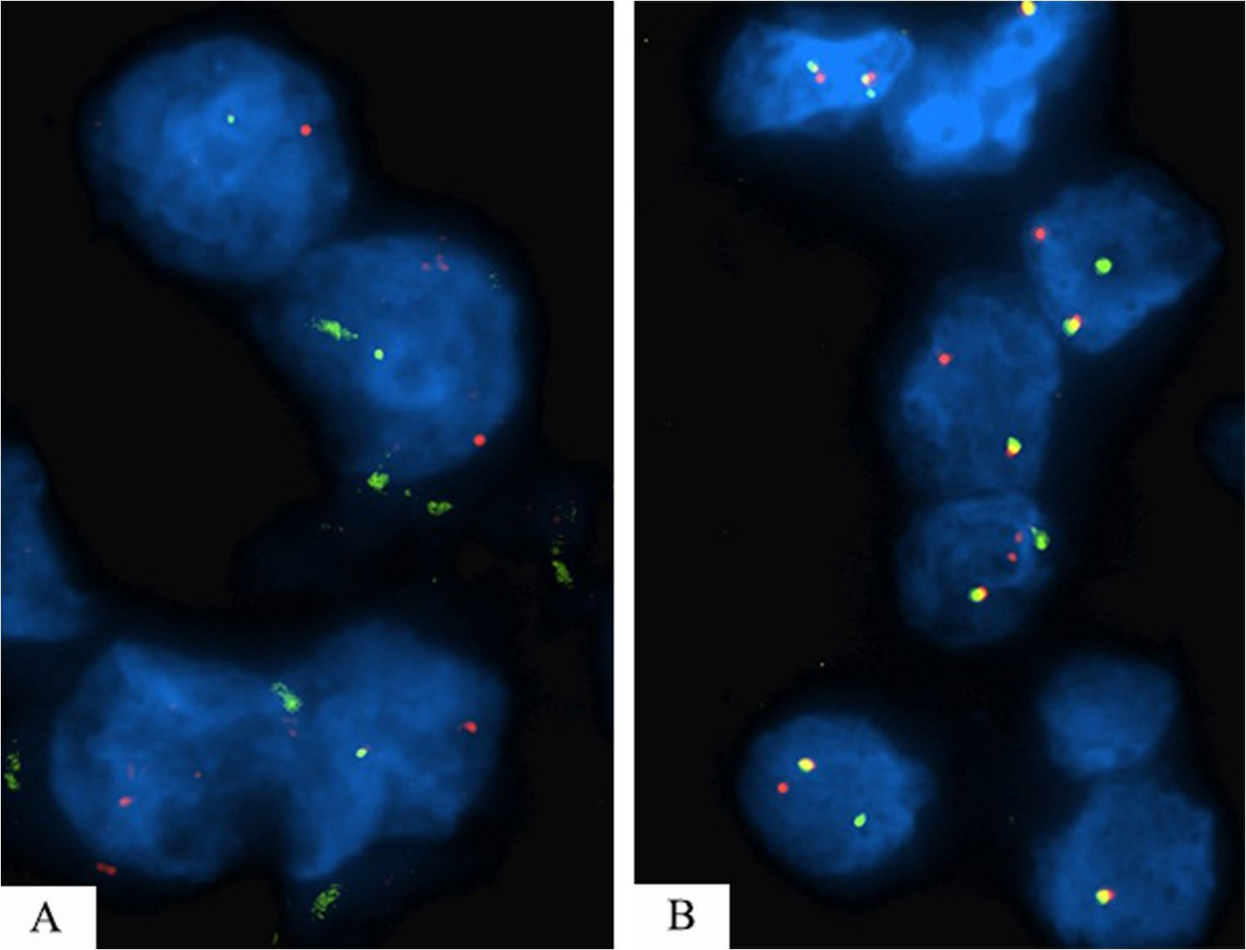
Fluorescence in situ hybridization of TFE3 (**A**) and TFEB (**B**) rearranged renal cell carcinoma. Distant red and green signals demonstrate the translocation by using a break-apart probe

Twenty-seven TFE3-rearranged renal cell carcinomas and ten TFEB-rearranged renal cell carcinomas were demonstrated by FISH analysis (Fig. 1). One hundred and fifty clear cell renal cell carcinomas, one hundred papillary renal cell carcinomas, fifty oncocytomas, fifty chromophobe renal cell carcinomas, and eighteen clear cell papillary renal cell tumors were also retrieved from the archives of the Pathology Department of Verona University and Pederzoli Hospital, Peschiera del Garda, Verona. All slides were reviewed by three experienced pathologists (AC, MB, and GM).

### Immunohistochemistry

Sections from tissue blocks of all the included types of renal cell carcinoma were immunohistochemically stained with the following antibodies: PAX8 (clone BC12; DSB), CD10 (clone 56C6, dilution 1:50; Novocastra), carbonic anhydrase 9 CA9 (polyclonal rabbit, dilution 1:1000; Abcam), cytokeratin 7 (clone RN7, dilution 1:100; Novocastra), alpha-methylacyl-CoA racemase AMACR (clone 13H7, dilution 1:25; Dako), S100A1 (clone M01, dilution 1:600; Abnova), parvalbumin (clone P19, dilution 1:500; Sigma), CD13 (clone 38C12, dilution 1:100; Novocastra), GATA3 (clone L50-823, dilution 1:150; BD Pharmingen), and cathepsin K (clone 3F9, dilution 1:2000; Abcam). To further analyze the immunohistochemical profile of MiT family translocation renal cell carcinomas, TFE3-rearranged-renal cell carcinomas and TFEB-rearranged renal cell carcinomas were also stained with HMB45 (dilution 1:30; Dako, Denmark), Melan-A (clone A103, dilution 1:50; Novocastra, UK), CD68 (clone PG-M1, dilution 1:50; Dako), cytokeratin 8–18 (clone 5D3, dilution 1:100; Novocastra), cytokeratin 20 (clone PW31, dilution 1:100; Novocastra), fumarate hydratase FH (clone J-13, dilution 1:50; Santa Cruz), and succinate dehydrogenase B SDHB (clone 21A11AE7, dilution 1:800, Abcam).

All samples were processed using a sensitive Bond Polymer Refine detection system in an automated Bond immunohistochemistry instrument (Leica Biosystems, Germany). The immunohistochemical expression of each marker for every tumor subtype was recorded. Concerning TFE3 and TFEB-rearranged renal cell carcinomas, three different cutoffs of expression were evaluated for each marker, respectively of 5%, 10%, and 20%.

### Statistical analysis

Fisher’s exact test was used to compare categorical data for immunohistochemical characteristics for each of above mentioned three different cutoff levels. All *P*-values were based on a two-tailed hypothesis. The results were considered statistically significant if the *P*-value was less than 0.05.

## Results

### MiT family translocation renal cell carcinomas (TFE3 and TFEB-rearranged renal cell carcinomas)

The pathological features of twenty-seven TFE3-rearranged renal cell carcinomas [41] and ten TFEB-rearranged renal cell carcinomas [19, 27, 35, 41, 42] have already been reported. The immunohistochemical results are here further expanded and detailed in Table 1 recording the percentage of expression.

**Table 1:** Immunohistochemical results of TFE3 and TFE3B-rearranged renal cell carcinomas of the present series

	Case	PAX 8	Cathepsin K	HMB45	MelanA	CD68(PG-M1)	CK8-18	CK7	CA9	PV	AMACR	CD10	CD13	SDHB	FH	GATA3	S100A1	CK20
TFE3	1	5% +	80% +	15% +	Neg	Neg	10% +	Neg	Neg	30% +	60% +	30% +	Neg	+	+	Neg	100% +	Neg
	2	80% +	100% +	Neg	70% +	Neg	Neg	Neg	Neg	1% +	10% +	40% +	80% +	+	+	Neg	100% +	Neg
	3	90% +	100% +	Neg	80% +	Neg	< 1% +	Neg	Neg	1% +	Neg	90% +	60% +	+	+	Neg	100% +	Neg
	4	5% +	100% +	Neg	Neg	Neg	Neg	Neg	5% +	Neg	60% +	90% +	30% +	+	+	Neg	100% +	Neg
	5	10%	90% +	Neg	Neg	Neg	N.A	N.A	N.A	N.A	N.A	N.A	N.A	N.A	N.A	N.A	N.A	N.A
	6	5% +	60% +	Neg	Neg	Neg	Neg	Neg	10% +	Neg	40% +	100% +	Neg	+	+	Neg	90% +	Neg
	7	5% +	100% +	5% +	Neg	Neg	< 5% +	Neg	< 1% +	Neg	Neg	90% +	5% +	+	+	Neg	100% +	Neg
	8	100% +	100% +	Neg	Neg	Neg	< 5% +	Neg	Neg	Neg	70% +	100% +	20% +	+	+	Neg	100% +	Neg
	9	100% +	100% +	Neg	10% +	Neg	20% +	Neg	Neg	Neg	5% +	100% +	60% +	+	+	Neg	100% +	Neg
	10	70% +	90% +	80% +	Neg	Neg	20% +	Neg	Neg	80% +	70% +	80% +	Neg	+	+	Neg	80% +	Neg
	11	60% +	30% +	5% +	Neg	Neg	5% +	5% +	10% +	Neg	60% +	60% +	Neg	+	+	Neg	100% +	Neg
	12	90% +	80% +	Neg	Neg	Neg	20% +	Neg	N.A	N.A	60% +	100% +	Neg	+	+	Neg	100% +	Neg
	13	10% +	Neg	Neg	Neg	Neg	Neg	Neg	5% +	Neg	70% +	60% +	5% +	+	+	Neg	100% +	Neg
	14	50% +	Neg	Neg	Neg	Neg	5% +	5% +	Neg	Neg	90% +	80% +	20% +	+	+	Neg	100% +	Neg
	15	90% +	Neg	N.A	N.A	Neg	Neg	Neg	1% +	N.A	90% +	N.A	Neg	+	+	Neg	N.A	N.A
	16	N.A	Neg	Neg	N.A	N.A	Neg	N.A	Neg	N.A	N.A	Neg	N.A	N.A	N.A	N.A	N.A	N.A
	17	100% +	Neg	Neg	Neg	N.A	Neg	Neg	< 5% +	Neg	90% +	90% +	90% +	+	+	Neg	5% +	Neg
	18	100% +	Neg	Neg	Neg	Neg	20% +	Neg	Neg	30% +	90% +	100% +	Neg	+	+	Neg	100% +	Neg
	19	30% +	Neg	1% +	Neg	Neg	40% +	1% +	5% +	30% +	40% +	50% +	40% +	+	+	Neg	60% +	Neg
	20	N.A	Neg	Neg	N.A	Neg	Neg	Neg	5% +	Neg	5–10% +	90% +	90% +	+	+	Neg	100% +	Neg
	21	90% +	Neg	Neg	Neg	N.A	N.A	Neg	Neg	2% +	90% +	90% +	Neg	+	+	Neg	90% +	Neg
	22	90% +	Neg	20% +	Neg	Neg	Neg		Neg	50% +	40% +	30% +	Neg	+	+	Neg	50% +	Neg
	23	100% +	Neg	30% +	Neg	Neg	Neg	Neg	Neg	Neg	5–10% +	100% +	Neg	+	+	Neg	Neg	Neg
	24	30% +	Neg	Neg	Neg	Neg	30% +	1% +	Neg	Neg	90% +	90% +	Neg	+	+	Neg	100% +	Neg
	25	50% +	100% +	5% +	5% +	N.A	Neg	Neg	Neg	N.A	5% +	N.A	N.A	N.A	N.A	N.A	N.A	N.A
	26	5%	1% +	Neg	Neg	Neg	N.A	Neg	1% +	Neg	20% +	30% +	Neg	N.A	N.A	N.A	5%	Neg
	27	1% +	5% +	Neg	Neg	Neg	90% +	2% +	1% +	Neg	60% +	N.A	N.A	N.A	N.A	N.A	Neg	Neg
TFEB	1	80% +	100% +	5% +	80% +	Neg	15% +	Neg	Neg	5% +	Neg	10% +	Neg	+	+	Neg	60% +	Neg
	2	80% +	70% +	5% +	80% +	Neg	30% +	Neg	Neg	Neg	Neg	Neg	Neg	+	+	Neg	60% +	Neg
	3	10% +	70% +	5% +	20% +	Neg	70% +	Neg	Neg	5% +	5% +	Neg	Neg	+	+	Neg	10% +	Neg
	4	70% +	100% +	5% +	80% +	Neg	30% +	Neg	Neg	5% +	5% +	10% +	5% +	+	+	Neg	100% +	Neg
	5	60% +	90% +	5% +	80% +	Neg	10% +	Neg	Neg	5% +	Neg	Neg	Neg	+	+	Neg	70% +	Neg
	6	20% +	80% +	10% +	80% +	Neg	5% +	Neg	Neg	Neg	Neg	5% +	Neg	+	+	Neg	10% +	Neg
	7	30% +	40% +	Neg	90% +	Neg	40% +	Neg	Neg	60% +	Neg	10% +	70% +	+	+	Neg	80% +	Neg
	8	60% +	< 5% +	20% +	10% +	Neg	90% +	10%	Neg	5% +	Neg	Neg	N.A	+	+	Neg	90% +	Neg
	9	< 1% +	80% +	1% +	60% +	Neg	30% +	Neg	Neg	20% +	5% +	Neg	Neg	+	+	Neg	60% +	Neg
	10	< 1% +	100% +	5% +	80% +	Neg	5% +	Neg	Neg	Neg	Neg	Neg	Neg	+	+	Neg	100% +	Neg

Abbreviations:* CA9* carbonic anhydrase 9, *PV* parvalbumin, *AMACR* alpha-methylacyl-CoA racemase, *SDHB* succinate dehydrogenase B, *FH* fumarate hydratase, *N.A*: no data available

Cathepsin K was observed in the most of MiT family translocation renal cell carcinomas (66% and 63% using the threshold of 5% and 10% or 20% positive cells respectively). About TFE3-rearranged renal cell carcinomas, immunolabeling was observed in roughly half of the cases (54% and 50% using the threshold of 5% and 10% or 20% positive cells respectively) whereas all the eleven TFEB-rearranged renal cell carcinomas evaluated stained positive for cathepsin K.

Melanocytic markers, HMB45 and Melan-A, were respectively positive in 15 of 36 (42%) and 14 of 34 (41%) of MiT family translocation renal cell carcinomas using the 5% cutoff. The percentage of cases considered positive for HMB45 drastically decreased when the higher cutoff was used (17% and 11% using the threshold of 10% and 20% respectively) while the percentage of positive cases was similar for Melan-A regardless of the cutoff (38% and 29% using the threshold of 10% and 20% respectively). TFEB-rearranged renal cell carcinomas were constantly immunolabeled for Melan-A (100%, 100%, and 90% of cases using the threshold of 5%, 10%, and 20% positive cells respectively), making such a reliable marker for the identification of these tumors, and frequently for HMB45 (80%, 20%, and 10% of the cases using the threshold of 5%, 10%, and 20% positive cells respectively). The expression of Melan-A and HMB45 was significantly lower in TFE3-rearranged renal cell carcinoma compared with TFEB-rearranged renal cell carcinoma, even when a 5% cutoff was used (17% and 27% respectively).

Regarding the proximal tubular markers such as CD10 and CD13, immunolabeling for the former was observed in 26 of 33 (79%), 25 of 33 (76%), and 22 of 33 (67%) of the MiT family translocation renal cell carcinomas, with the respective increasing cutoffs. As for TFE3-rearranged renal cell carcinomas, among 23 tumors, CD10 stained positive in 22 of the cases (96%), regardless of the cutoff considered, whereas it was positive just in 4 (40%), 3 (30%), and none (0%) of all the ten cases of TFEB-rearranged renal cell carcinomas, respectively, using a 5%, 10%, and 20% positivity threshold. CD13 expression, instead, was found in 13 of 32 (41%) and 10 of 32 (31%) MiT family translocation renal cell carcinomas tested, respectively, with a 5% and both a 10% and 20% cutoff. Among TFE3-rearranged renal cell carcinomas, 11 of 23 tumors (48%), considering a 5% cutoff, and 9 of 23 tumors (39%), both with a 10% and a 20% threshold, were positive for CD13; a weak expression of the same marker was instead noticed for TFEB-rearranged renal cell carcinomas, with only 2 (22%) and 1 (11%) of 9 tumors staining positive, employing a 5% and both a 10% and 20% respectively considered.

As for the distal tubular markers such as GATA3 and parvalbumin, none of the MiT family translocation renal cell carcinomas retrieved was positive for the former. About parvalbumin expression, among 33 MiT family translocation renal cell carcinomas 12 (36%), using a 5% cutoff, and 7 (21%), both considering a 10% and 20% cutoff, labeled positive for it. While 5 of 22 TFE3-rearranged renal cell carcinomas showed positivity for such marker in more than 20% of the cells (22%), the remaining 17 cases were completely negative or patchy positive for it. Moreover, 7 of 10 (70%) and 2 of 10 (20%) TFEB-rearranged renal cell carcinomas revealed positive parvalbumin expression respectively considering a 5% threshold and both a 10% and a 20% threshold.

Considering other markers commonly evaluated when dealing with renal cell carcinomas, CA9 was expressed by 6 of 35 (17%), 2 of 35 (6%), and none of 35 (0%) MiT family translocation renal cell carcinomas, respectively, using a 5%, 10%, and 20% cutoff. Namely, whereas CA9 immunolabeling was found in 6 of 25 (24%) TFE3-rearranged renal cell carcinomas with a 5% threshold, in 2 of them (8%) and none of them (0%), respectively, referring to a 10% and 20% cutoff, all the TFEB-rearranged renal cell carcinomas were negative for such marker. Among the 35 MiT family translocation renal cell carcinomas tested, AMACR, instead, stained positive in 26 (74%), 19 (54%), and 18 cases (51%), regarding a 5%, 10%, and 20% cutoff respectively. Positive expression was found in 23 of 25 (92%), 19 of 25 (76%), and 18 of 25 (72%) TFE3-rearranged renal cell carcinomas, using the same thresholds. Nevertheless, only 3 of 10 (30%) TFEB-rearranged renal cell carcinomas were considered positive for AMACR when a 5% cutoff was used, while none of them showed positive staining in more than 10% of the cells.

Furthermore, S100A1 was typically positive and CK7 was usually negative regardless of the cutoff used either considering the overall MiT family translocation renal cell carcinomas or TFE3 and TFEB-rearranged renal cell carcinoma separately. Regarding CK8-18 immunolabeling instead, it was noticed in 19 of 34 (56%), 15 of 34 (44%), and 13 of 34 (38%) MiT family translocation renal cell carcinomas, using a 5%, 10%, and 20% cutoff respectively. While the expression is lower for TFE3-rearranged renal cell carcinomas (41%, 33%, and 29% respectively with a 5%, a 10%, and 20% threshold), most of TFEB-rearranged renal cell carcinomas labeled positive for CK8-18 (100%, 80%, and 60% respectively with 5%, 10%, and 20% threshold).

None of the MiT family translocation renal cell carcinomas considered expressed CD68 (PG-M1) neither CK20. SDHB and FH were retained in all the cases tested.

The immunohistochemical results of clear cell renal cell carcinoma, papillary renal cell carcinoma, clear cell papillary renal cell tumor, chromophobe renal cell carcinoma, and oncocytoma are detailed in supplementary Table 1.

### Comparison of immunohistochemical markers and statistical relevance

#### MiT family translocation renal cell carcinoma versus clear cell renal cell carcinoma

Regardless of the cutoff used, in the differential diagnosis with clear cell renal cell carcinoma, a statistically significant correlation was found with MiT family translocation renal cell carcinomas and negative expression of CA9 (*p* = 0.0001) and CD13 (*p* = 0.0001), with MiT family translocation renal cell carcinomas and positive expression of AMACR (*p* = 0.0001), cathepsin K (*p* = 0.0001), and parvalbumin (*p* = 0.0001 with a 5% cutoff and *p* = 0.0007 with both a 10% and a 20% cutoff).

As far as TFE3-rearranged renal cell carcinomas were concerned, despite the cutoff taken into account, a negative expression of CA9 (*p* = 0.0001) and CD13 (*p* = 0.0003 with a 5% cutoff and *p* = 0.0001 with both a 10% and a 20% cutoff) statistically correlated with the diagnosis of such tumor subtype, as well as cathepsin K (*p* = 0.0001), AMACR (*p* = 0.0001), and parvalbumin (*p* = 0.0021) positivity.

About TFEB-rearranged renal cell carcinomas, finally, a statistical correlation of relevant levels was reported with negative expression of CD10 (*p* = 0.0002 with a 5% cutoff and *p* = 0.0001 with both a 10% and a 20% cutoff), CA9 (*p* = 0.0002 with both a 5% and a 10% cutoff and *p* = 0.0001 with both a 20% cutoff), and CD13 (*p* = 0.0001) and with cathepsin K (*p* = 0.0001) and parvalbumin positivity (*p* = 0.0001 with a 5% threshold and *p* = 0.0048 with both a 10% and a 20% threshold), no matter what the cutoff used was.

##### Useful tools

**Fig. 2:**
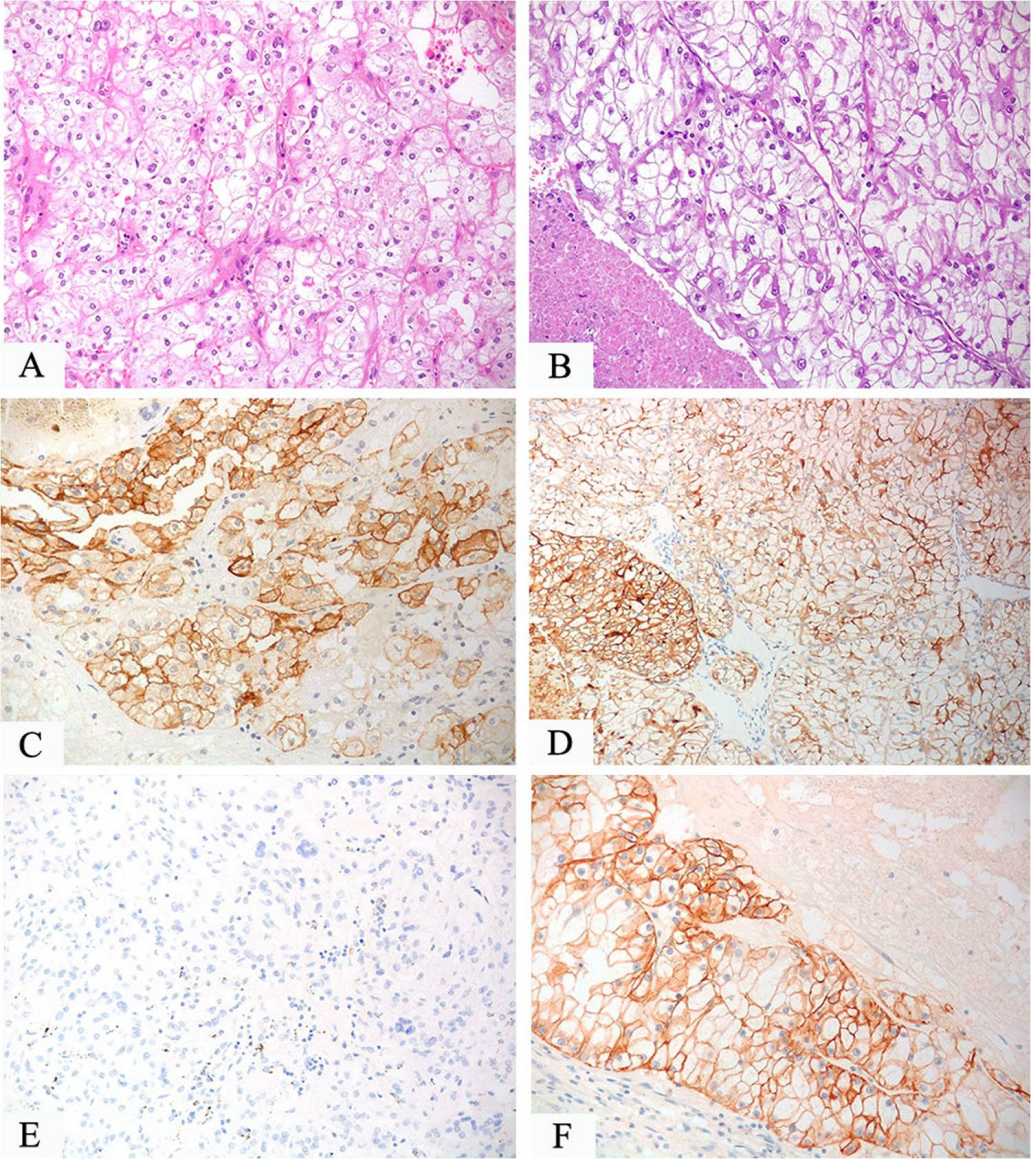
**A** TFE3-rearranged renal cell carcinoma composed of cells with clear cytoplasm (**A**) and an example of clear cell renal cell carcinoma arranged in nests (**B**). Both tumors are positive for CD10 (**C**, **D**), whereas CA9 is negative in TFE3-rearranged renal cell carcinoma (**E**) typically expressed in clear cell carcinoma (**F**)

CD10 is not useful in the differential diagnosis between clear cell renal cell carcinoma and TFE3-rearranged renal cell carcinoma since both tumors are usually labeled for this marker, but it helps distinguish from TFEB-rearranged renal cell carcinoma. CA9 is usually negative either in TFE3 or TFEB-rearranged renal cell carcinoma whereas it is an important positive reliable marker in clear cell renal cell carcinoma. On the other hand, cathepsin K is positive in TFEB-rearranged renal cell carcinoma and half of TFE3-rearranged renal cell carcinoma while it is negative in clear cell renal cell carcinoma (Fig. 2).

##### Suggested panel

Suggested panel is as follows: CA9 and cathepsin K.

#### MiT family translocation renal cell carcinoma versus clear cell papillary renal cell tumor

When referring to clear cell papillary renal cell tumor, there was a strong statistically significant correlation, despite the threshold of positivity considered, with MiT family translocation renal cell carcinomas and negative expression of CA9 (*p* = 0.0001), CK7 (*p* = 0.0001), and GATA3 (*p* = 0.0001), along with positive expression of CD10 (*p* = 0.0001 with both a 5% and 10% cutoff and *p* = 0.0011 with a 20% cutoff), AMACR (*p* = 0.0001 with both a 5% and 10% cutoff and *p* = 0.0002 with a 20% cutoff), and cathepsin K (*p* = 0.0001). Similar results were observed when TFE3-rearranged renal cell carcinoma was considered. However, in TFEB-rearranged renal cell carcinoma, CD10 and AMACR staining are not statistically correlated (*p* = 0.5 and *p* = 1 respectively).

##### Useful tools

**Fig. 3:**
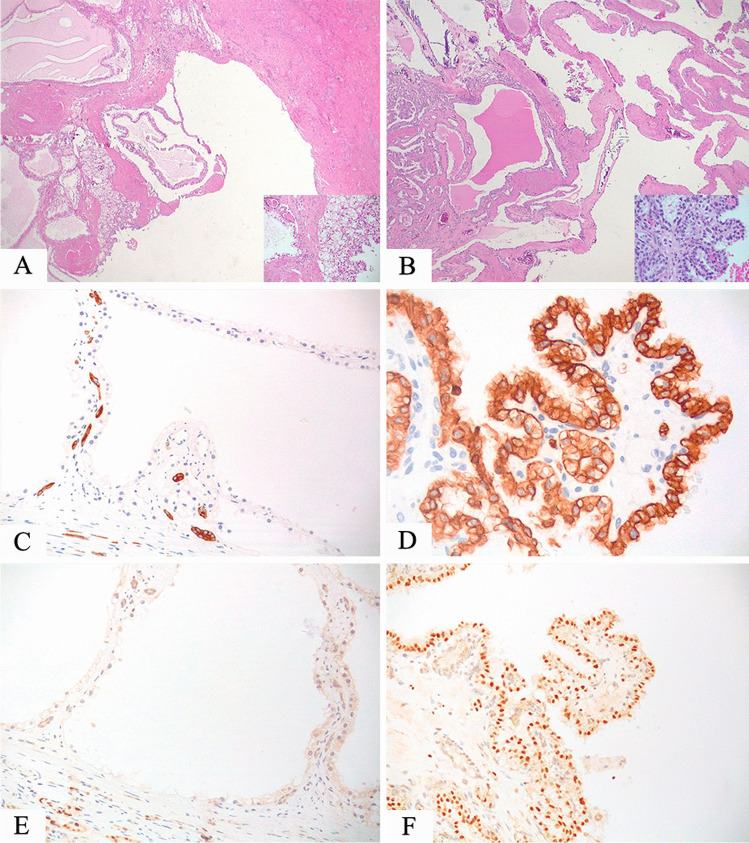
**A** cystic TFE3-rearranged renal cell carcinoma lined by cells with clear cytoplasm (the inset underlining the nuclei oriented toward the lumen) (**A**), is negative for CK7 (**C**) and GATA3 (**E**). Conversely, a clear cell papillary renal cell tumor nearly entirely cystic (the inset highlighting a partially papillary architecture) (**B**), showing CK7 (**D**) and GATA3 (**F**) positivity

GATA3, CK7, and CA9 are the most reliable markers in this differential diagnosis since are positive in clear cell papillary renal cell tumor and negative in TFE3 and TFEB-rearranged. CD10 is not useful in the differential diagnosis between clear cell papillary renal cell tumor and TFEB-rearranged renal cell carcinoma since both tumors are typically negative for this marker, but it helps distinguish from TFE3-rearranged renal cell carcinoma, usually positive. Cathepsin K is positive in TFEB-rearranged renal cell carcinoma and half of TFE3-rearranged renal cell carcinoma while it is negative in clear cell papillary renal cell tumor (Fig. 3).

##### Suggested panel

Suggested panel is as follows: CA9, CK7, GATA3, and cathepsin K.

#### MiT family translocation renal cell carcinoma versus papillary renal cell carcinoma

As for the differential diagnosis with papillary renal cell carcinoma, despite what cutoff was used, a significant statistical correlation was noticed with MiT family translocation renal cell carcinomas and negative expression of CK7 (*p* = 0.0001), AMACR (*p* = 0.066 with a 5% cutoff and *p* = 0.0001 with both a 10% and 20% cutoff), and CD13 (*p* = 0.0001 with both a 5% and 10% cutoff and *p* = 0.0002 with a 20% cutoff) and with MiT family translocation renal cell carcinomas and positive expression of parvalbumin (*p* = 0.0001 with a 5% threshold and *p* = 0.0095 with a 10% and 20% threshold) and cathepsin K (*p* = 0.0001).

Speaking of TFE3-rearranged renal cell carcinomas, regardless the cutoff, a negative expression of only CK7 (*p* = 0.0001) and CD13 (*p* = 0.0018 with a 5% cutoff, *p* = 0.0002 with a 10% cutoff and *p* = 0.0001 with a 20% one) was of statistically significant value, as well as positive immunolabeling for CD10 (*p* = 0.0002), parvalbumin (*p* = 0.0016), and cathepsin K (*p* = 0.0001). Furthermore, negative expression of CA9 (*p* = 0.017) and AMACR (*p* = 0.0045), both considering a 20% positivity cutoff, and positive staining for S100A1 (*p* = 0.0282), with the lowest cutoff of 5%, was significantly strong alike.

A statistically relevant correlation was also noted between TFEB-rearranged renal cell carcinomas and negativity for CK7 (*p* = 0.0001), AMACR (*p* = 0.0001), and CD13 (*p* = 0.0003 with a 5% threshold and *p* = 0.0001 with both a 10% and a 20% threshold) along with their positive expression of cathepsin K (*p* = 0.0001), no matter what cutoff value was considered.

##### Useful tools

**Fig. 4:**
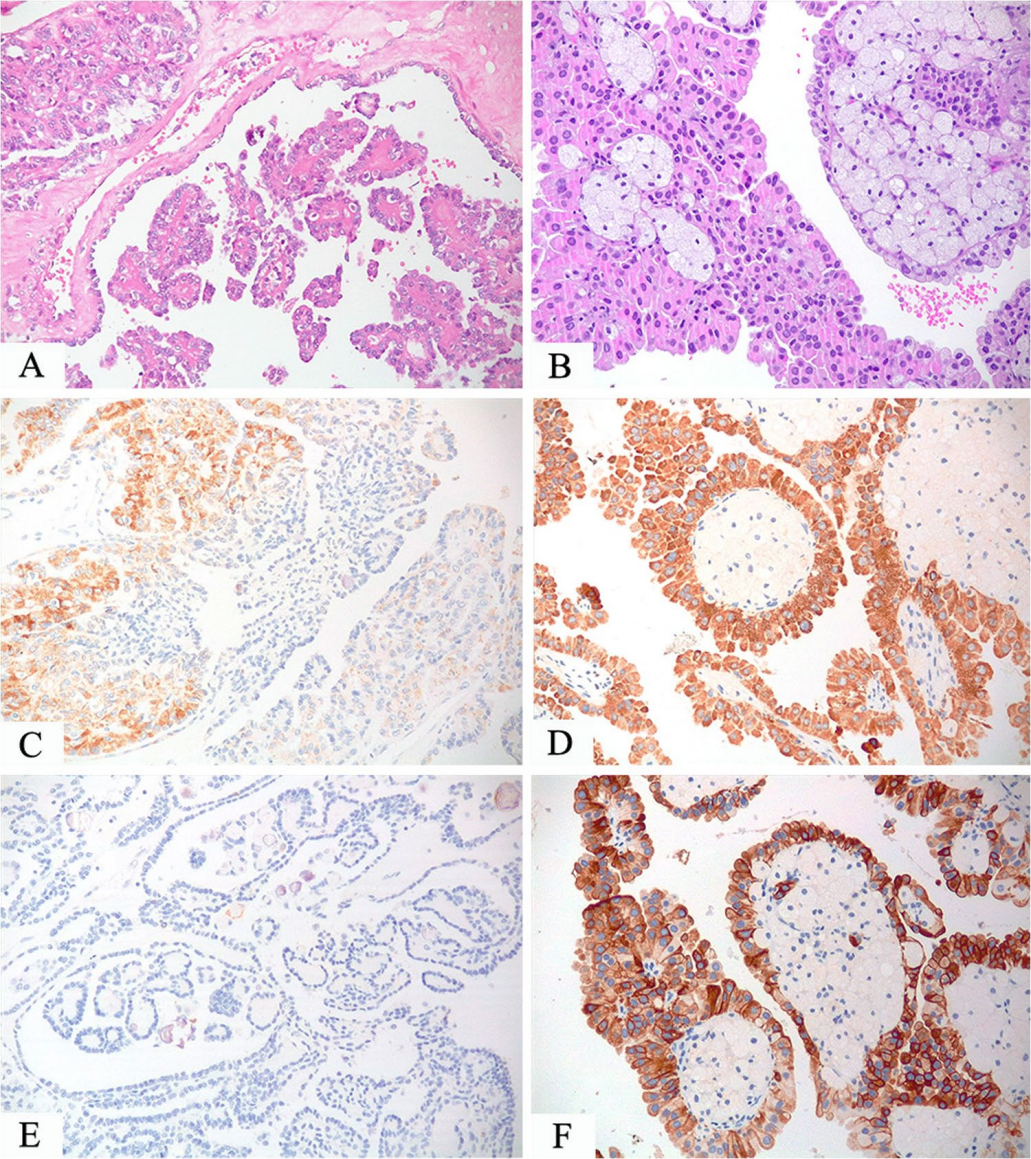
TFE3-rearranged renal cell carcinoma with papillary architecture (**A**) and papillary renal cell carcinoma (**B**). Both tumors expressed AMACR (**C**, **D**) which is not useful in this differential diagnosis. Staining for CK7 is negative in TFE3-rearranged renal cell carcinoma (**E**) but positive in papillary renal cell carcinoma (**F**)

AMACR is not useful in the differential diagnosis between papillary renal cell carcinoma and TFE3-rearranged renal cell carcinoma since both tumors are usually labeled for this marker, but it helps distinguish from TFEB-rearranged renal cell carcinoma. CK7 is usually negative either in TFE3 or TFEB-rearranged renal cell carcinoma whereas it is an important positive reliable marker in papillary renal cell carcinoma. On the other hand, cathepsin K is positive in TFEB-rearranged renal cell carcinoma and half of TFE3-rearranged renal cell carcinoma while it is negative in papillary renal cell carcinoma (Fig. 4).

##### Suggested panel

Suggested panel is as follows CK7 and cathepsin K.

#### MiT family translocation renal cell carcinoma versus chromophobe renal cell carcinoma

In cases with histological features recalling that of chromophobe carcinomas, negative expression of CK7 (*p* = 0.0001) and parvalbumin (*p* = 0.0001), along with immunolabeling for CD10 (*p* = 0.0001), AMACR (*p* = 0.0001), S100A1 (*p* = 0.0001), CD13 (*p* = 0.0001 with a 5% threshold and *p* = 0.0008 with both a 10% and 20% threshold), and cathepsin K (*p* = 0.0001) were statistically consistent with the diagnosis of MiT family translocation renal cell carcinomas, all these results observed concerning each of the above-mentioned positivity cutoffs.

TFE3-rearranged renal cell carcinomas revealed the same results. In addition, interestingly, positive staining for CA9 was also found to be statistically relevant, but only when a 5% positivity cutoff was considered (*p* = 0.0239).

Finally, as far as TFEB-rearranged renal cell carcinomas were concerned, negativity for CK7 (*p* = 0.0159 with both a 5% and 10% cutoff and *p* = 0.0014 with a 20% one) and parvalbumin (*p* = 0.0183 with a 5% threshold and *p* = 0.0001 with both a 10% and 20% one) as well as positive expression of S100A1 (*p* = 0.0001) and cathepsin K (*p* = 0.0001) statistically correlated with the diagnosis of such tumor subtype, despite the cutoff chosen. On the other hand, no relevant statistical correlation was found with immunohistochemical expression of either CD10, AMACR, or CD13, in contrast with the data observed with TFE3-rearranged renal cell carcinomas.

##### Useful tools

CK7, S100A1, and parvalbumin are helpful immunohistochemical markers in the differential diagnosis between chromophobe renal cell carcinoma and TFE3/TFEB-rearranged renal cell carcinoma. Expression of CK7 and parvalbumin along with the absence of S100A1 is characteristic of chromophobe renal cell carcinoma, whereas strong and diffuse labeling of S100A1 along with the under-expression of CK7 and parvalbumin are typical of TFE3/TFEB-rearranged renal cell carcinoma. Cathepsin K is positive in TFEB-rearranged renal cell carcinoma and half of TFE3-rearranged renal cell carcinoma while it is negative in chromophobe renal cell carcinoma.

##### Suggested panel

Suggested panel is as follows: CK7, S100A1, parvalbumin, and cathepsin K.

#### MiT family translocation renal cell carcinoma versus oncocytoma

Negative expression of parvalbumin (*p* = 0.0001) and positive staining for CD10 (*p* = 0.0001 with a 5% cutoff, *p* = 0.0004 with a 10% cutoff, and *p* = 0.0070 with a 20% cutoff), AMACR (*p* = 0.0001), CD13 (*p* = 0.0001), and cathepsin K (*p* = 0.0001) were strongly statistically consistent with the diagnosis of MiT family translocation renal cell carcinoma despite what positivity threshold was used. Similar results were also noted for negative expression of S100A1 (*p* = 0.0305) and CK7 (*p* = 0.0364), respectively referring to a 10% and 20% cutoff.

The analysis from TFE3-rearranged renal cell carcinomas data revealed similar results, whereas, regarding TFEB-rearranged renal cell carcinomas, cathepsin K positive staining was the only immunohistochemical result of statistical significance for all the positivity thresholds considered (*p* = 0.0001) and a statistical correlation was also collected with parvalbumin negativity (*p* = 0.0001), even though only with a 5% and 10% positivity threshold.

##### Useful tools

**Fig. 5:**
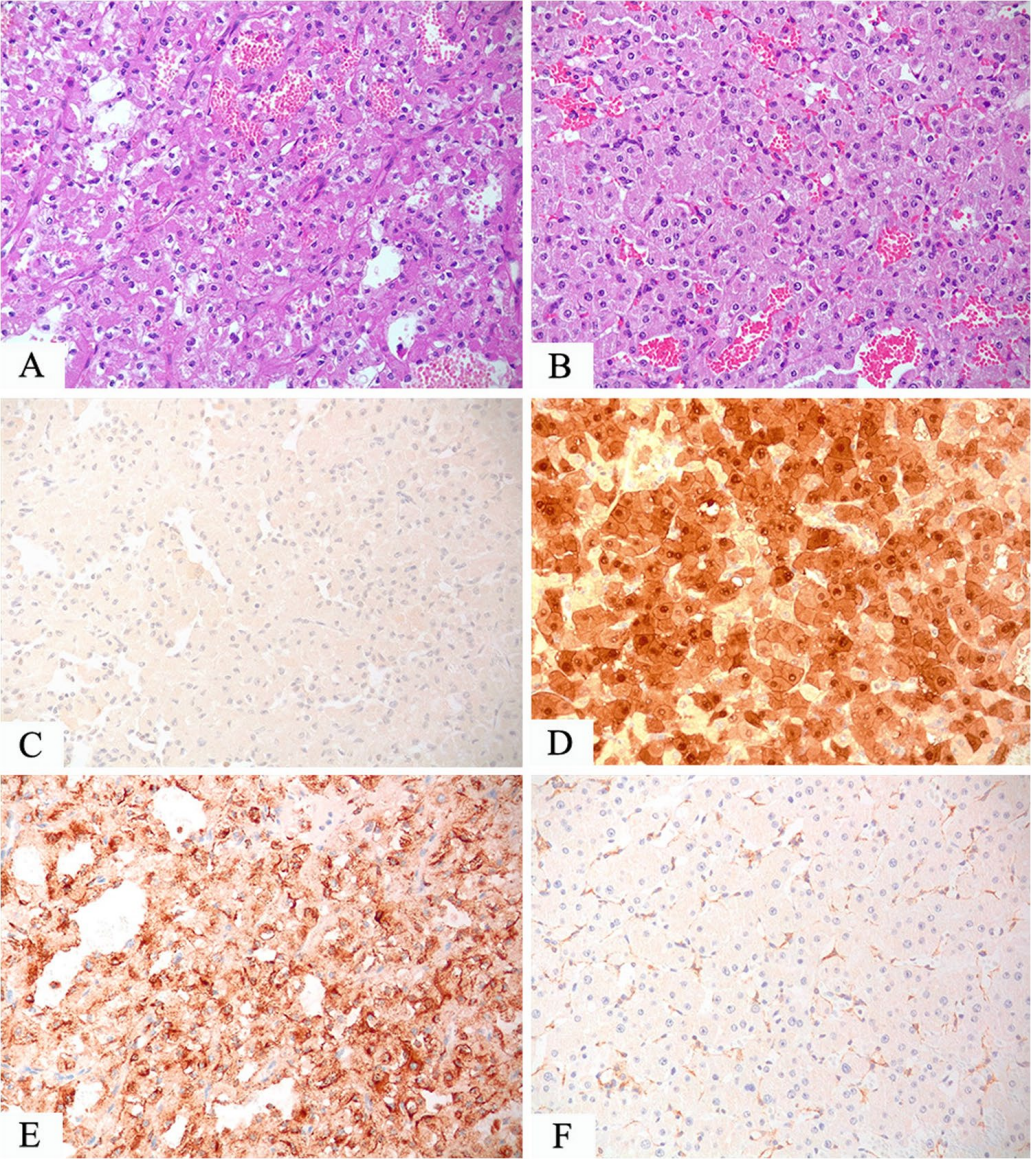
TFEB-rearranged renal cell carcinoma made up of round cells with granular eosinophilic cytoplasm (**A**) negative for parvalbumin (**C**) and diffusely positive for cathepsin K (**E**). A classic example of oncocytoma (**B**) expressing parvalbumin (**D**) and negative for cathepsin K (**F**)

Cathepsin K and parvalbumin are the two useful markers in the differential diagnosis between oncocytoma and TFE3/TFEB-rearranged renal cell carcinoma (Fig. 5).

##### Suggested panel

Suggested panel is as follows: parvalbumin and cathepsin K.

In Tables 2, 3, and 4, the comparison for the immunohistochemical markers with the *p*-value among MiT family translocation renal cell carcinomas and renal cell tumors is shown, respectively. using a 5%, 10%, and 20% positivity threshold.

**Table 2:** Comparison for the immunohistochemical markers with the *p* value using a 5% positivity threshold

a. Immunohistochemical comparison of TFE3-rearranged renal cell carcinoma									
	*CD10*	*CD13*	*CK7*	*AMACR*	*CA9*	*GATA3*	*S100A1*	*PV*	*Cathepsin K*
Clear cell RCC	*p* = 0.47	***p* = 0.0003**	*p* = 0.6924	***p* = 0.0001**	***p* = 0.0001**	*p* = 1.000	*p* = 0.1971	***p* = 0.0021**	***p* = 0.0001**
Clear cell papillary RCT	***p* = 0.0001**	*p* = 0.5302	***p* = 0.0001**	***p* = 0.0001**	***p* = 0.0001**	***p* = 0.0001**	*p* = 0.0999	*p* = 0.2980	***p* = 0.0005**
Papillary RCC	***p* = 0.0002**	***p* = 0.0018**	***p* = 0.0001**	*p* = 1.000	*p* = 0.2245	*p* = 1.000	***p* = 0.0282**	***p* = 0.016**	***p* = 0.0001**
Oncocytoma	***p* = 0.0001**	***p* = 0.0001**	*p* = 0.7090	***p* = 0.0001**	*p* = 0.2478	*p* = 0.5289	*p* = 1.000	***p* = 0.0001**	***p* = 0.0001**
Chromophobe RCC	***p* = 0.0001**	***p* = 0.0001**	***p* = 0.0001**	***p* = 0.0001**	***p* = 0.0239**	*p* = 0.1541	***p* = 0.0001**	***p* = 0.0001**	***p* = 0.0001**
b. Immunohistochemical comparison of TFEB-rearranged renal cell carcinoma									
	*CD10*	*CD13*	*CK7*	*AMACR*	*CA9*	*GATA3*	*S100A1*	*PV*	*Cathepsin K*
Clear cell RCC	***p* = 0.0002**	***p* = 0.0001**	*p* = 0.5675	*p* = 0.3819	***p* = 0.0002**	*p* = 1.000	*p* = 0.0659	***p* = 0.0001**	***p* = 0.0001**
Clear cell papillary RCT	*p* = 0.3746	*p* = 0.6692	***p* = 0.0001**	*p* = 0.0504	***p* = 0.0001**	***p* = 0.0074**	***p* = 0.0411**	***p* = 0.013**	***p* = 0.0001**
Papillary RCC	*p* = 0.2053	***p* = 0.0003**	***p* = 0.0001**	***p* = 0.0001**	*p* = 0.196	*p* = 1.000	***p* = 0.0152**	***p* = 0.0001**	***p* = 0.0001**
Oncocytoma	*p* = 1.000	***p* = 0.0292**	*p* = 1.000	***p* = 0.0067**	*p* = 0.5615	*p* = 1.000	*p* = 0.3322	*p* = 0.1044	***p* = 0.0001**
Chromophobe RCC	*p* = 0.2057	*p* = 0.0818	***p* = 0.0159**	*p* = 0.1227	*p* = 1.000	*p* = 0.5707	***p* = 0.0001**	***p* = 0.0183**	***p* = 0.0001**
c. Immunohistochemical comparison of MiT family translocation renal cell carcinoma									
	*CD10*	*CD13*	*CK7*	*AMACR*	*CA9*	*GATA3*	*S100A1*	*PV*	*Cathepsin K*
Clear cell RCC	*p* = 0.0986	***p* = 0.0001**	*p* = 0.7255	***p* = 0.0001**	***p* = 0.0001**	*p* = 0.5807	***p* = 0.0378**	***p* = 0.0001**	***p* = 0.0001**
Clear cell papillary RCT	***p* = 0.0001**	*p* = 0.7715	***p* = 0.0001**	***p* = 0.0001**	***p* = 0.0001**	***p* = 0.0001**	***p* = 0.0252**	***p* = 0.0204**	***p* = 0.0001**
Papillary RCC	*p* = 0.0643	***p* = 0.0001**	***p* = 0.0001**	***p* = 0.0066**	*p* = 0.0797	*p* = 0.5567	***p* = 0.0019**	***p* = 0.0001**	***p* = 0.0001**
Oncocytoma	***p* = 0.0001**	***p* = 0.0001**	*p* = 0.7272	***p* = 0.0001**	*p* = 0.0997	*p* = 0.4965	*p* = 0.5143	***p* = 0.0001**	***p* = 0.0001**
Chromophobe RCC	***p* = 0.0001**	***p* = 0.0001**	***p* = 0.0001**	***p* = 0.0001**	*p* = 0.0825	*p* = 0.0628	***p* = 0.0001**	***p* = 0.0001**	***p* = 0.0001**

Abbreviations:* RCC* renal cell carcinoma, *RCT* renal cell tumor, *AMACR* alpha-methylacyl-CoA racemase, *CA9* carbonic anhydrase 9, *PV* parvalbumin

The bold means statistically significant *p*-value

**Table 3:** Comparison for the immunohistochemical markers with the *p* value using a 10% positivity threshold

a. Immunohistochemical comparison of TFE3-rearranged renal cell carcinoma									
	*CD10*	*CD13*	*CK7*	*AMACR*	*CA9*	*GATA3*	*S100A1*	*PV*	*Cathepsin K*
Clear cell RCC	*p* = 0.47	***p* = 0.0001**	*p* = 0.3613	*p* = 0.5317	***p* = 0.0001**	*p* = 1.000	*p* = 0.7988	***p* = 0.0021**	***p* = 0.0001**
Clear cell papillary RCT	***p* = 0.0001**	*p* = 1.000	***p* = 0.0001**	***p* = 0.0001**	***p* = 0.0001**	***p* = 0.0001**	*p* = 0.2749	*p* = 0.2980	***p* = 0.0012**
Papillary RCC	***p* = 0.0002**	***p* = 0.0002**	***p* = 0.0001**	*p* = 0.2450	*p* = 0.2245	*p* = 1.000	*p* = 0.2232	***p* = 0.016**	***p* = 0.0001**
Oncocytoma	***p* = 0.0001**	***p* = 0.0001**	*p* = 0.09	***p* = 0.0001**	*p* = 1.000	*p* = 0.5289	*p* = 0.5089	***p* = 0.0001**	***p* = 0.0001**
Chromophobe RCC	***p* = 0.0001**	***p* = 0.0002**	***p* = 0.0001**	***p* = 0.0001**	*p* = 1.000	*p* = 0.1541	***p* = 0.0001**	***p* = 0.0001**	***p* = 0.0001**
b. Immunohistochemical comparison of TFEB-rearranged renal cell carcinoma									
	*CD10*	*CD13*	*CK7*	*AMACR*	*CA9*	*GATA3*	*S100A1*	*PV*	*Cathepsin K*
Clear cell RCC	***p* = 0.0001**	***p* = 0.0001**	*p* = 0.5675	*p* = 0.3613	***p* = 0.0002**	*p* = 1.000	*p* = 0.0659	***p* = 0.0048**	***p* = 0.0001**
Clear cell papillary RCT	*p* = 0.6457	*p* = 0.3642	***p* = 0.0001**	*p* = 1.000	***p* = 0.0001**	***p* = 0.0074**	***p* = 0.0411**	*p* = 0.2174	***p* = 0.0001**
Papillary RCC	*p* = 0.06	***p* = 0.0001**	***p* = 0.0001**	***p* = 0.0001**	*p* = 0.196	*p* = 1.000	***p* = 0.0152**	*p* = 0.1273	***p* = 0.0001**
*Oncocytoma*	*p* = 1.000	0.1930	*p* = 1.000	*p* = 1.000	*p* = 0.5615	*p* = 1.000	*p* = 0.3322	***p* = 0.0001**	***p* = 0.0001**
*Chromophobe RCC*	*p* = 0.4065	*p* = 0.3571	***p* = 0.0159**	*p* = 0.5773	*p* = 1.000	*p* = 0.5707	***p* = 0.0001**	***p* = 0.0001**	***p* = 0.0001**
c. Immunohistochemical comparison of MiT family translocation renal cell carcinoma									
	*CD10*	*CD13*	*CK7*	*AMACR*	*CA9*	*GATA3*	*S100A1*	*PV*	*Cathepsin K*
Clear cell RCC	*p* = 0.0544	***p* = 0.0001**	*p* = 0.6962	***p* = 0.0001**	***p* = 0.0001**	*p* = 0.5807	*p* = 0.1808	***p* = 0.0007**	***p* = 0.0001**
Clear cell papillary RCT	***p* = 0.0001**	*p* = 0.7635	***p* = 0.0001**	***p* = 0.0001**	***p* = 0.0001**	***p* = 0.0001**	*p* = 0.1075	*p* = 0.1648	***p* = 0.0001**
Papillary RCC	*p* = 0.1071	***p* = 0.0001**	***p* = 0.0001**	***p* = 0.0001**	*p* = 0.0797	*p* = 0.5567	***p* = 0.0181**	***p* = 0.0095**	***p* = 0.0001**
Oncocytoma	***p* = 0.0004**	***p* = 0.0001**	*p* = 0.2302	***p* = 0.0001**	*p* = 0.6610	*p* = 0.4965	***p* = 0.0305**	***p* = 0.0001**	***p* = 0.0001**
Chromophobe RCC	***p* = 0.0001**	***p* = 0.0008**	***p* = 0.0001**	***p* = 0.0001**	*p* = 1.000	*p* = 0.0628	***p* = 0.0001**	***p* = 0.0001**	***p* = 0.0001**

Abbreviations: *RCC* renal cell carcinoma, *RCT* renal cell tumor, *AMACR* alpha-methylacyl-CoA racemase, *CA9* carbonic anhydrase 9, *PV* parvalbumin

The bold means statistically significant *p*-value

**Table 4:** Comparison for the immunohistochemical markers with the *p* value using a 20% positivity threshold

a. Immunohistochemical comparison of TFE3-rearranged renal cell carcinoma									
	*CD10*	*CD13*	*CK7*	*AMACR*	*CA9*	*GATA3*	*S100A1*	*PV*	*Cathepsin K*
Clear cell RCC	*p* = 0.47	***p* = 0.0001**	*p* = 0.3613	***p* = 0.0001**	***p* = 0.0001**	*p* = 1000	*p* = 0.7988	***p* = 0.0021**	***p* = 0.0001**
Clear cell papillary RCT	***p* = 0.0001**	*p* = 1.000	***p* = 0.0001**	***p* = 0.0001**	***p* = 0.0001**	***p* = 0.0001**	*p* = 0.2749	*p* = 0.2980	***p* = 0.0012**
Papillary RCC	***p* = 0.0002**	***p* = 0.0002**	***p* = 0.0001**	***p* = 0.0045**	***p* = 0.017**	*p* = 1000	*p* = 0.2232	***p* = 0.016**	***p* = 0.0001**
Oncocytoma	***p* = 0.0001**	***p* = 0.0001**	*p* = 0.09	***p* = 0.0001**	*p* = 0.2478	*p* = 0.5289	*p* = 0.5089	***p* = 0.0001**	***p* = 0.0001**
Chromophobe RCC	***p* = 0.0001**	***p* = 0.0002**	***p* = 0.0001**	***p* = 0.0001**	*p* = 0.5125	*p* = 0.1541	***p* = 0.0001**	***p* = 0.0001**	***p* = 0.0001**
b. Immunohistochemical comparison of TFEB-rearranged renal cell carcinoma									
	*CD10*	*CD13*	*CK7*	*AMACR*	*CA9*	*GATA3*	*S100A1*	*PV*	*Cathepsin K*
Clear cell RCC	***p* = 0.0001**	***p* = 0.0001**	*p* = 1.000	*p* = 0.3611	***p* = 0.0001**	*p* = 1.000	*p* = 0.7293	***p* = 0.0048**	***p* = 0.0001**
Clear cell papillary RCT	*p* = 0.5053	*p* = 0.2021	***p* = 0.0001**	*p* = 1.000	***p* = 0.0001**	***p* = 0.0074**	*p* = 0.3864	*p* = 0.2174	***p* = 0.0001**
Papillary RCC	***p* = 0.0001**	***p* = 0.0001**	***p* = 0.0001**	***p* = 0.0001**	*p* = 0.196	*p* = 1.000	*p* = 0.3248	*p* = 0.1273	***p* = 0.0001**
Oncocytoma	***p* = 0.0251**	*p* = 0.1930	*p* = 0.58	*p* = 1.000	*p* = 0.5615	*p* = 1.000	*p* = 0.6595	***p* = 0.0001**	***p* = 0.0001**
Chromophobe RCC	*p* = 0.3301	*p* = 0.3571	***p* = 0.0014**	*p* = 0.5773	*p* = 1.000	*p* = 0.5707	***p* = 0.0001**	***p* = 0.0001**	***p* = 0.0001**
c. Immunohistochemical comparison of MiT family translocation renal cell carcinoma									
	*CD10*	*CD13*	*CK7*	*AMACR*	*CA9*	*GATA3*	*S100A1*	*PV*	*Cathepsin K*
Clear cell RCC	***p* = 0.0032**	***p* = 0.0001**	*p* = 0.2154	***p* = 0.0001**	***p* = 0.0001**	*p* = 0.5807	*p* = 0.5160	***p* = 0.0007**	***p* = 0.0001**
Clear cell papillary RCT	***p* = 0.0011**	*p* = 0.7592	***p* = 0.0001**	***p* = 0.0002**	***p* = 0.0001**	***p* = 0.0001**	*p* = 0.2631	*p* = 0.1648	***p* = 0.0001**
Papillary RCC	*p* = 0.5453	***p* = 0.0002**	***p* = 0.0001**	***p* = 0.0001**	***p* = 0.0037**	*p* = 0.5567	*p* = 0.0927	***p* = 0.0095**	***p* = 0.0001**
Oncocytoma	***p* = 0.0070**	***p* = 0.0001**	***p* = 0.0364**	***p* = 0.0001**	*p* = 0.0997	*p* = 0.4965	*p* = 0.5566	***p* = 0.0001**	***p* = 0.0001**
Chromophobe RCC	***p* = 0.0001**	***p* = 0.0008**	***p* = 0.0001**	***p* = 0.0001**	*p* = 0.4937	*p* = 0.0628	***p* = 0.0001**	***p* = 0.0001**	***p* = 0.0001**

Abbreviations: *RCC* renal cell carcinoma, *RCT* renal cell tumor, *AMACR* alpha-methylacyl-CoA racemase, *CA9* carbonic anhydrase 9, *PV* parvalbumin

## Discussion

Despite initially considered rare tumors, TFE3 and TFEB-rearranged renal cell carcinoma represent 1–4% of renal cell carcinomas diagnosed among adults [13]. Proper identification of these tumor types is challenging since histological features may be ambiguous and often overlap with other more common types of renal cell neoplasm. In general, TFE3 and TFEB-rearranged renal cell carcinomas ought to be considered in the differential diagnosis, especially in young patients, every time pathologists have to deal with a renal tumor showing unusual microscopic findings [13, 29]. To support the diagnosis, *TFE3/TFEB* gene translocation should be demonstrated by FISH break-apart assay or the gene fusion identified by RNA sequencing [43]. Nevertheless, these techniques are not available in all laboratories and a thorough evaluation of their immunophenotype might be worth it for correctly identifying them. Moreover, in the last years, increasing use of immunohistochemistry has been observed due to the new clinic-pathologic entities identified.

Several studies addressed the immunohistochemical profile to aid in the classcarcinoma [44–46]. Staining for cathepsin K, CA9, CK7, and HMB45 has been claimed as helpful for this purpose. Basically, translocation renal cell carcinoma is labeled for cathepsin K and HMB45 while it is negative for CA9 and CK7. Moreover, previous manuscripts mainly focused on the differential diagnosis between TFE3-rearranged renal cell carcinoma and clear cell renal cell carcinoma [44, 46]. As previously pointed out, TFE3-rearranged renal cell carcinoma is likely to be misdiagnosed as clear cell renal cell carcinoma if a restricted immunohistochemical panel is applied. In this scenario, CD10 is not helpful regardless of the threshold of positivity considered, whereas performing cathepsin K and CA9 is useful in sorting the diagnostic quandary out. However, staining for CD10 has an important value in the differential diagnosis with TFEB-rearranged renal cell carcinoma, along with cathepsin K and CA9.

Besides clear cell renal cell carcinoma, other tumors can be misclassified. TFE3-rearranged renal cell carcinoma may also resemble clear cell papillary renal cell tumor. Those neoplasms usually labeled for CK7, CA9, and GATA3, not expressed in TFE3-rearranged renal cell carcinomas, which are instead commonly positive for cathepsin K. Either TFE3-rearranged renal cell carcinomas or TFEB-rearranged renal cell carcinomas demonstrating papillary architecture can be confused with papillary renal cell carcinoma. While AMACR is not useful in the differential diagnosis with TFE3-rearranged renal cell carcinomas, being positive in both tumors, such marker is usually under-expressed in TFEB-rearranged renal cell carcinomas. Of course, staining for CK7 favors papillary renal cell carcinoma, confirming its usefulness in distinguishing papillary renal cell carcinoma from TFE3 and TFEB-rearranged renal cell carcinomas. When TFE3 and TFEB-rearranged renal cell carcinomas exhibit extensive eosinophilic features, they can mimic oncocytoma and the eosinophilic variant of chromophobe renal cell carcinoma. To distinguish them, cathepsin K and parvalbumin are very helpful, in addition to CK7 and S100A1 for chromophobe renal cell carcinoma [47, 48]. Nevertheless, cathepsin K is expressed in other oncocytic neoplasms, such as the recently described eosinophilic solid and cystic renal cell carcinoma [49, 50]. These tumors may show overlapping morphological and immunohistochemical features with MiT family translocation renal cell carcinoma, especially with TFEB-rearranged renal cell carcinoma, both positive for cathepsin K and Melan-A. Although not addressed in the present manuscript, eosinophilic solid and cystic renal cell carcinomas immunolabeled for CK20 [51, 52], unlike TFEB-rearranged renal cell carcinoma, which has been claimed as a reliable marker in the differential diagnosis. However, conflicting data have been reported about the role of CK20 as a key diagnostic marker [53], so molecular analysis by FISH assay looking for *TFEB* gene alterations is still warranted in the most controversial cases.

Another important aspect of this study is the proper cutoff employed to define an immunohistochemical marker as positive since positive/negative results can be considered differently among pathologists. As different thresholds of positivity have been reported, it is important to establish if there are variances in results using several cutoffs of expression to consider an immunohistochemical marker as positive or negative. As expected, the threshold for positivity established is important for HMB45 and Melan-A. Even a patchy expression should be considered a positive result, especially for TFE3-rearranged renal cell carcinoma in which melanogenesis markers are less consistent. On the other hand, the different percentages (> 5%, > 10%, > 20%) of neoplastic cells labeling for cathepsin K do not change the result since the expression is usually strong and diffuse [54, 55]. Interestingly, some *p*-values of the different immunohistochemical markers vary based on the different percentages (> 5%, > 10%, > 20%). For instance, in the differential diagnosis between papillary renal cell carcinoma and TFE3-rearranged renal cell carcinoma, AMACR results are statistically significant when used 20% of positivity as a threshold whereas its expression is not significant with lower cutoffs, supporting the hypothesis that it is better to consider a lower percentage of positivity. On the other side, S100A1 seems an important marker in the differential diagnosis between TFEB-rearranged renal cell carcinoma and papillary renal cell carcinoma (*p* = 0.0152) using 5% and 10% of positivity whereas it is not significant with higher cutoffs, suggesting to use the high threshold of positivity. Overall, aside from melanogenesis markers and AMACR, in clinical practice, it is better to use a higher threshold to consider a positive immunohistochemical marker.

Finally, the study highlighted the importance, also from an immunohistochemical point of view, to maintain TFE3 and TFEB-rearranged renal cell carcinoma as distinct entities, as recommended by the forthcoming WHO classification of renal tumors. When grouped together under the heading MiT family translocation renal cell carcinoma, the value of some immunohistochemical markers changed dramatically. For instance, staining for CD10 which is usually observed in TFE3-rearranged renal cell carcinoma and under-expressed in TFEB-rearranged renal cell carcinoma seems significant in the differential diagnosis of MiT family translocation renal cell carcinoma and clear cell renal cell carcinoma. However, as abovementioned, CD10 is not useful in this differential diagnosis when we are dealing with TFE3-rearranged renal cell carcinoma.

In conclusion, the threshold to define a positive immunohistochemical result is not univocal but depends on the immunohistochemical marker considered. For instance, any expression of melanogenesis markers should be considered positive. A panel of immunohistochemistry markers useful to distinguish TFE3/TFEB-rearranged renal cell carcinoma from other common renal cell neoplasms should include cathepsin K, CA9, CK7, and parvalbumin (Table 5). In this scenario, tumors with equivocal morphological features and doubtful immunoprofile should be analyzed by FISH, which reliably detect the most common rearrangements. However, subtle *TFE3* gene inversions, such as *RBM10* and *NONO* gene fusions, may lead to false negative results by FISH and require further molecular analysis.

**Table 5:** The immunohistochemical panel to differentiate TFE3-TFEB rearranged RCC from common renal cell neoplasms

	CA9	CK7	Cathepsin K	PV
Clear cell RCC	+	Neg	Neg	Neg
Papillary RCC	Neg	+	Neg	Neg
Clear cell papillary *RCT*	+	+	Neg	Neg
Chromophobe RCC	Neg	+	Neg	+
Oncocytoma	Neg	Neg	Neg	+
TFE3-rearranged RCC	Neg	Neg	±	Neg
TFEB-rearranged RCC	Neg	Neg	+	Neg

Abbreviations: *RCC* renal cell carcinoma, *RCT* renal cell tumor, *CA9* carbonic anhydrase 9, *PV* parvalbumin

## Supplementary Information

Below is the link to the electronic supplementary material.

Supplementary file1 (DOCX 17 KB)
