# Supplementary material for: TFE3 and TFEB-rearranged renal cell carcinomas: an immunohistochemical panel to differentiate from common renal cell neoplasms
Source: Virchows Arch. 2022 Aug 18;481(6):877–91. doi: 10.1007/s00428-022-03380-x (PMC9734233; doi:10.1007/s00428-022-03380-x)
Supplement: Supplementary file 1 — Supplementary file1 (DOCX 17 KB) [file 428_2022_3380_MOESM1_ESM.docx]

**Table S1.** The immunohistochemical results of clear cell renal cell carcinoma, papillary renal cell carcinoma, clear cell papillary tumor, chromophobe renal cell carcinomas, and oncocytomas of the present series.

|  | Clear cell RCC | | Papillary RCC | | Clear cell papillary RCT | | Chromophobe RCC | | Oncocytoma | |  |  |
| --- | --- | --- | --- | --- | --- | --- | --- | --- | --- | --- | --- | --- |
|  | Positive N (%) | Negative | Positive | Negative | Positive | Negative | Positive | Negative | Positive | Negative |  |  |
| Cathepsin K | 0 (0%) | 132 (100%) | 0 (0%) | 81 (100%) | 0 (0%) | 14 (100%) | 0 (0%) | 41 (100%) | 0 (0%) | 41 (100%) |  |  |
| CK7 | 10 (7%) | 134 (93%) | 81 (85%) | 14 (15%) | 18 (100%) | 0 (0%) | 39 (80%) | 10 (20%) | 6 (12%) | 43 (88%) |  |  |
| CA9 | 141 (96%) | 6 (4%) | 13 (19%) | 56 (81%) | 16 (100%) | 0 (0%) | 1 (3%) | 38 (97%) | 1 (1%) | 32 (99%) |  |  |
| PV | 3 (2%) | 127 (98%) | 4 (4%) | 86 (90%) | 0 (0%) | 12 (100%) | 47 (94%) | 3 (6%) | 48 (96%) | 2 (4%) |  |  |
| AMACR | 19 (15%) | 108 (85%) | 89 (93%) | 7 (7%) | 0 (0%) | 17 (100%) | 4 (9%) | 42 (91%) | 0 (0%) | 43 (100%) |  |  |
| CD10 | 124 (89%) | 16 (11%) | 55 (59%) | 39 (41%) | 3 (17%) | 15 (83%) | 8 (16%) | 41 (84%) | 16 (35%) | 29 (65%) |  |  |
| CD13 | 116 (82%) | 25 (18%) | 77 (79%) | 20 (21%) | 3 (17%) | 15 (83%) | 1 (2%) | 44 (98%) | 0 (0%) | 46 (100%) |  |  |
| GATA3 | 1 (1%) | 128 (99%) | 3 (4%) | 82 (96%) | 13 (87%) | 2 (13%) | 5 (12%) | 38 (88%) | 2 (5%) | 38 (95%) |  |  |
| S100A1 | 96 (74%) | 34 (26%) | 59 (65%) | 32 (35%) | 8 (62%) | 5 (38%) | 3 (6%) | 44 (94%) | 43 (86%) | 7 (14%) |  |  |

Abbreviations: RCC: renal cell carcinoma, RCT: renal cell tumor, CA9: carbonic anhydrase 9, PV: parvalbumin, AMACR: alpha-methylacyl-CoA racemase.
